# Supplementary material for: Molecular Activation of the Kv11.1 Channel Reprograms EMT in Colon Cancer by Inhibiting TGFβ Signaling via Activation of Calcineurin
Source: Cancers (Basel). 2021 Nov 30;13(23):6025. doi: 10.3390/cancers13236025 (PMC8656647; doi:10.3390/cancers13236025)
Supplement: Supplementary file 1 [file cancers-13-06025-s001.zip › cancers-1434748 supplementary Figure S1 Original Western blots .pdf]

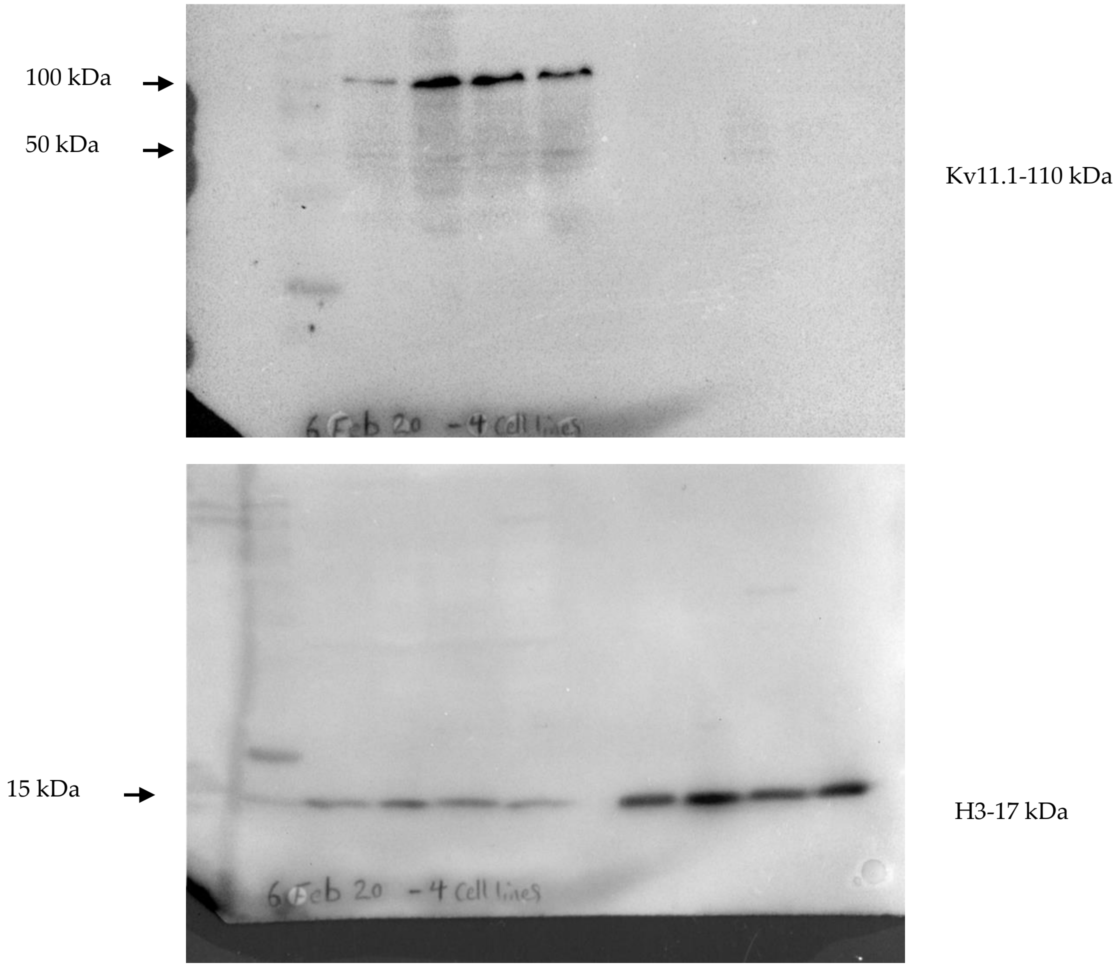
100 kDa
50 kDa
Kv11.1-110 kDa
6 Feb 20 - 4 cell lines
15 kDa
H3-17 kDa
6 Feb 20 - 4 cell lines

Figure 1. (A)

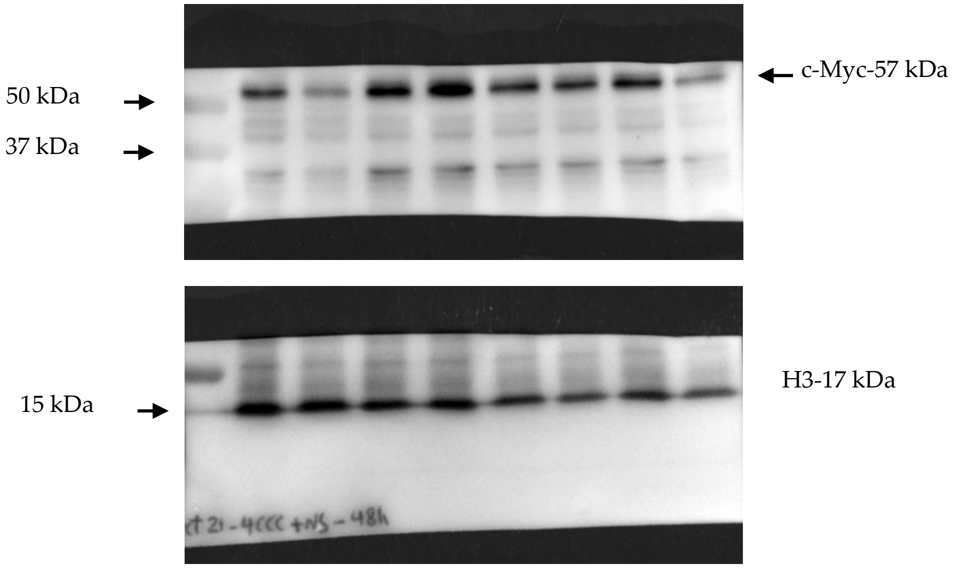

Figure 2. (B)

	HT29-DMSO	HT29-NS1643	SW480-DMSO	SW480-NS1643	FET-DMSO	FET-NS1643
c-Myc	0.69	0.23	1.24	1.20	1.13	0.32

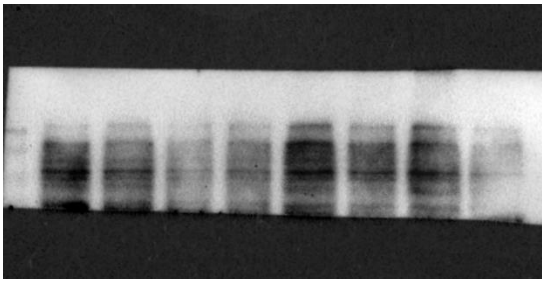

Short exposure time

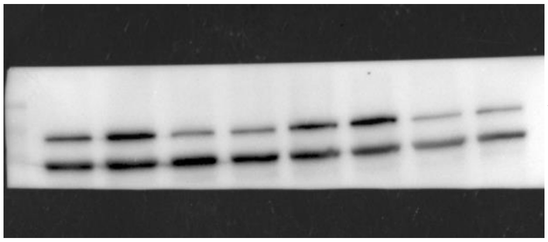

Long exposure time for FET cells

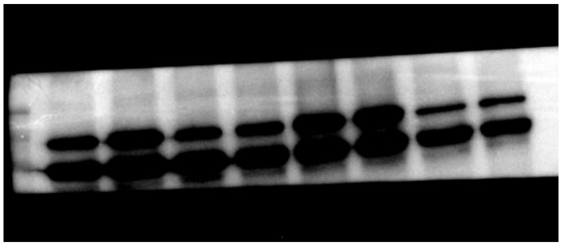

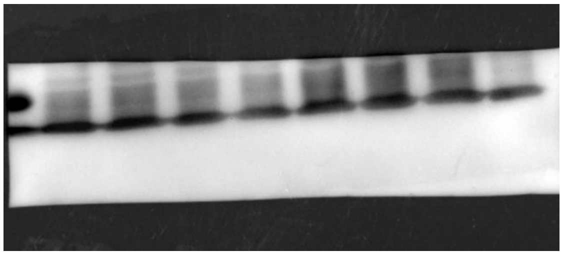

Figure 3. (C)

	HT29-DMSO	HT29-NS1643	SW480-DMSO	SW480-NS1643	FET-DMSO	FET-NS1643
N -Cadherin	0.19	0.13	0.15	0.35	0.10	0.04
E -Cadherin	0.33	0.65	0.24	0.27	0.14	0.27

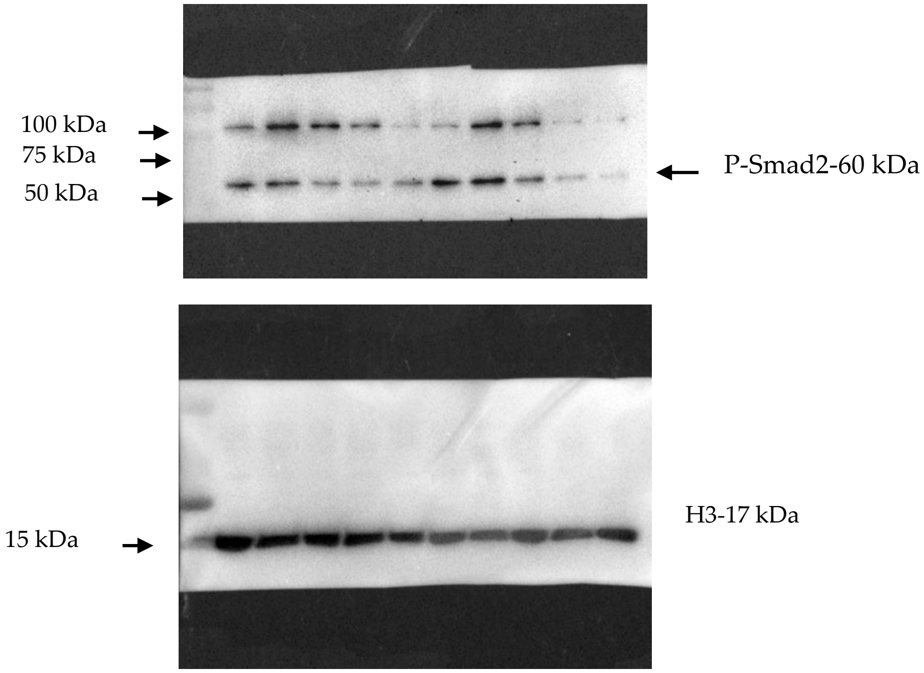

Figure 4.(A) and (C)

	HT29-DMSO 2hr	HT29-NS1643 2hr	FET-DMSO 2hr	FET-NS1643 2hr
P-Smad2	0.25	0.10	0.45	0.13

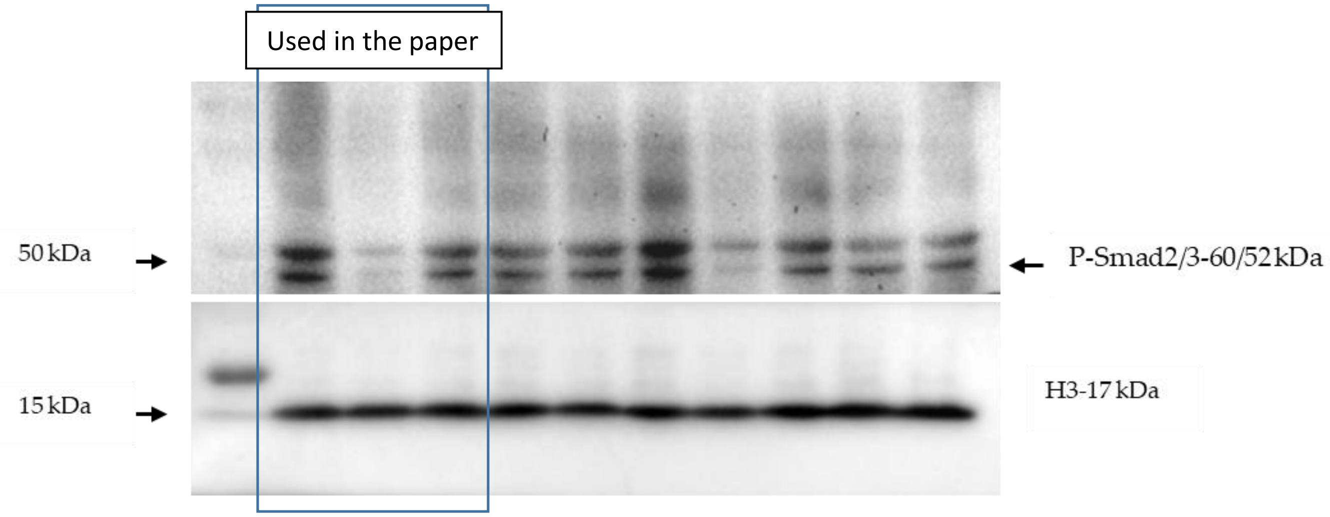

Figure 4. (D)

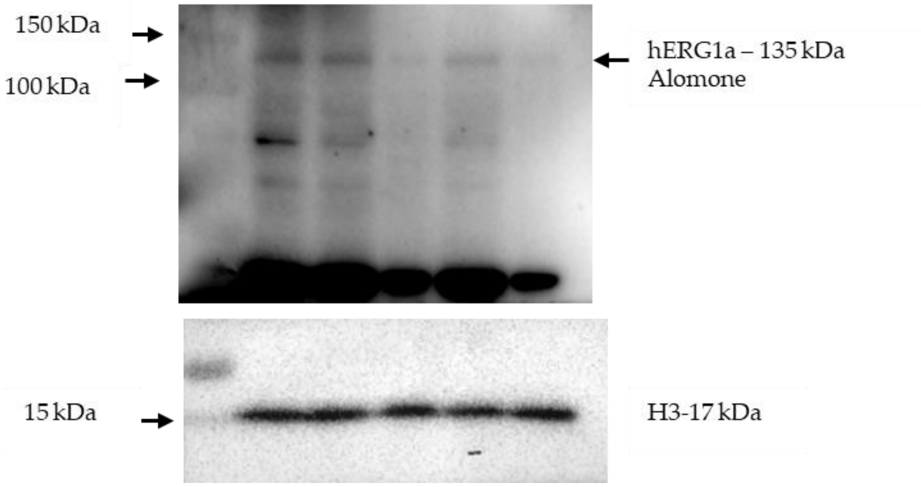

	Control	NS1643 20 min	SiRNA+NS1643 20 min
P-Smad2	30.9	9.3	19.9
P-Smad3	26.7	1.9	11.7

Figure 4. (D)

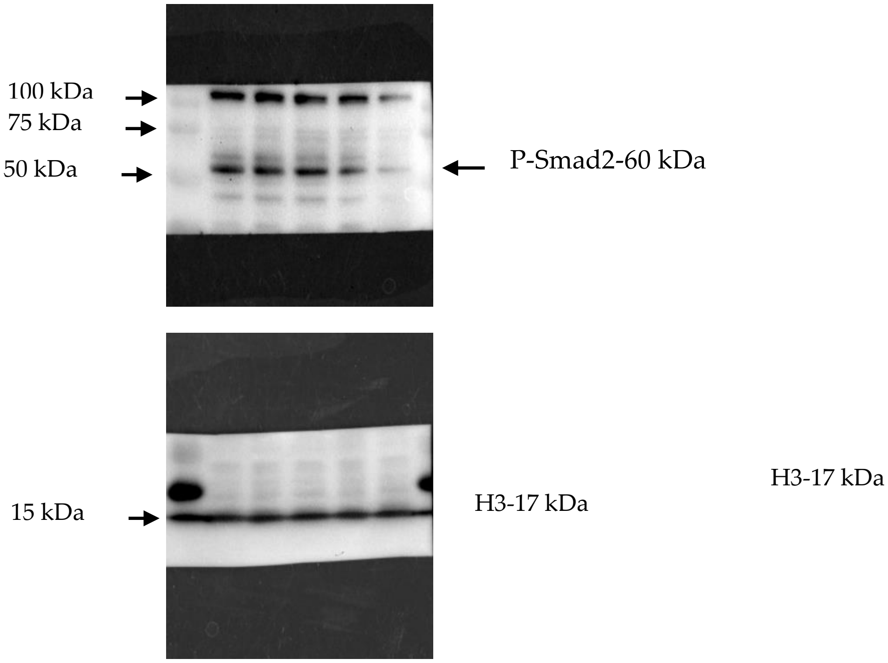
H3-17 kDa

Figure 4. (B)

	SW480-DMSO 2hr	SW480-NS1643 2hr
P-Smad2	0.32	0.21

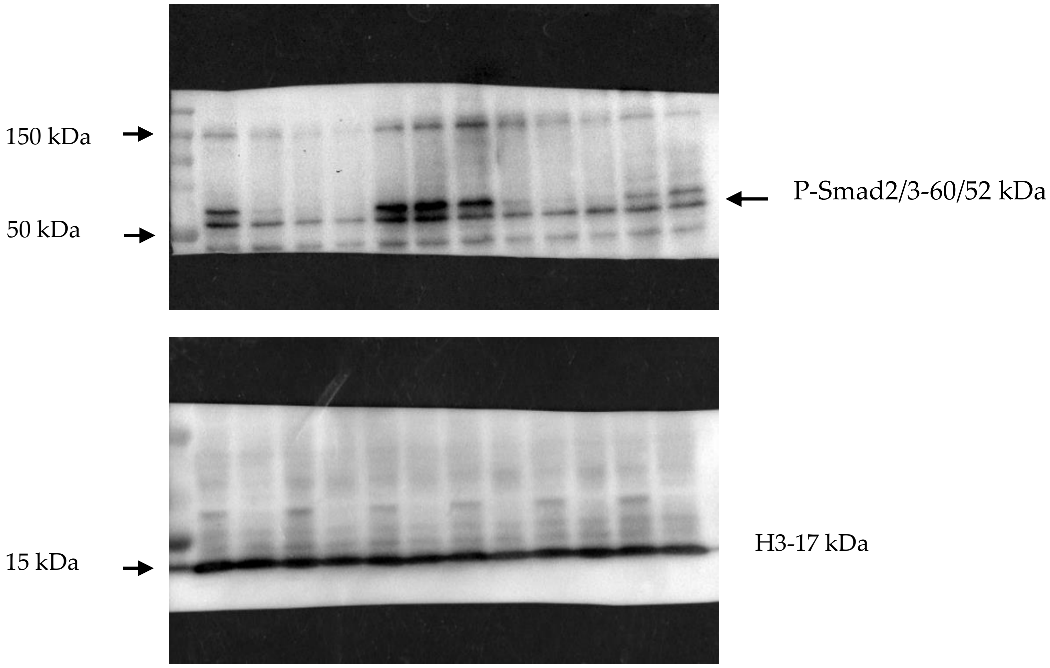

Figure 5. (**A**)

	Control	NS1643 2hr	TGFβ 2hr	TGFβ-NS1643 2hr
P-Smad2	0.15	0.02	0.20	0.02
P-Smad3	0.16	0.07	0.19	0.14

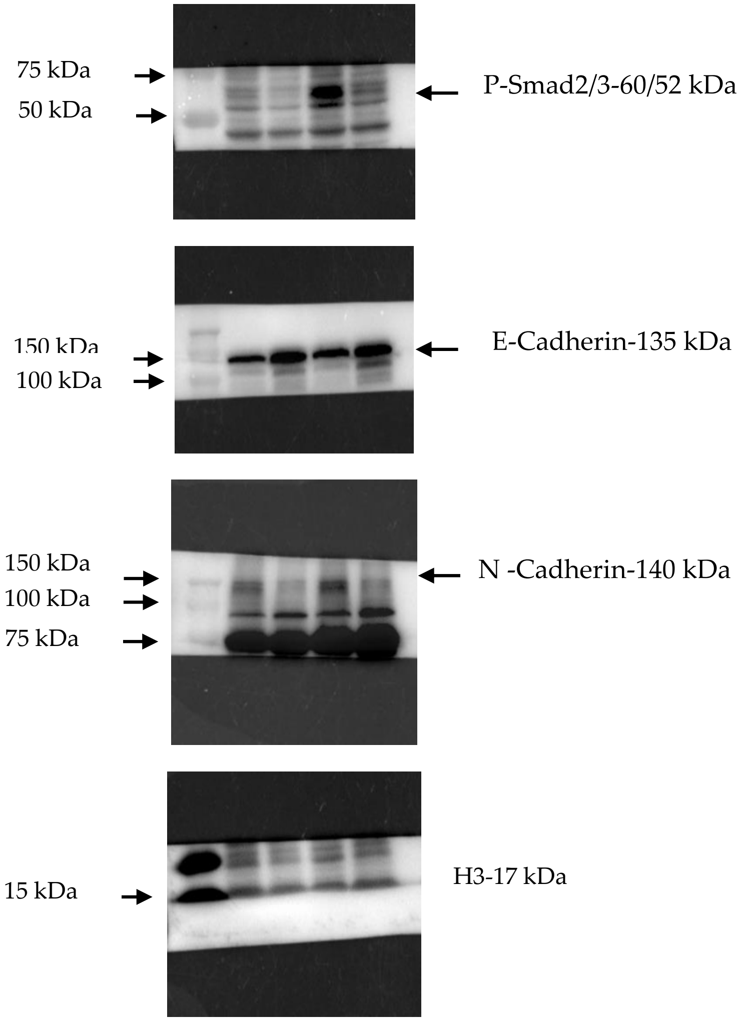

Figure 5. (**B**)

	Control	NS1643	TGFβ	TGFβ-NS1643
P-Smad2	0.013	0.004	0.414	0.029
P-Smad3	0.061	0.043	0.108	0.048
E-Cadherin	0.36	0.72	0.50	0.75
N-Cadherin	0.05	0.01	0.30	0.01

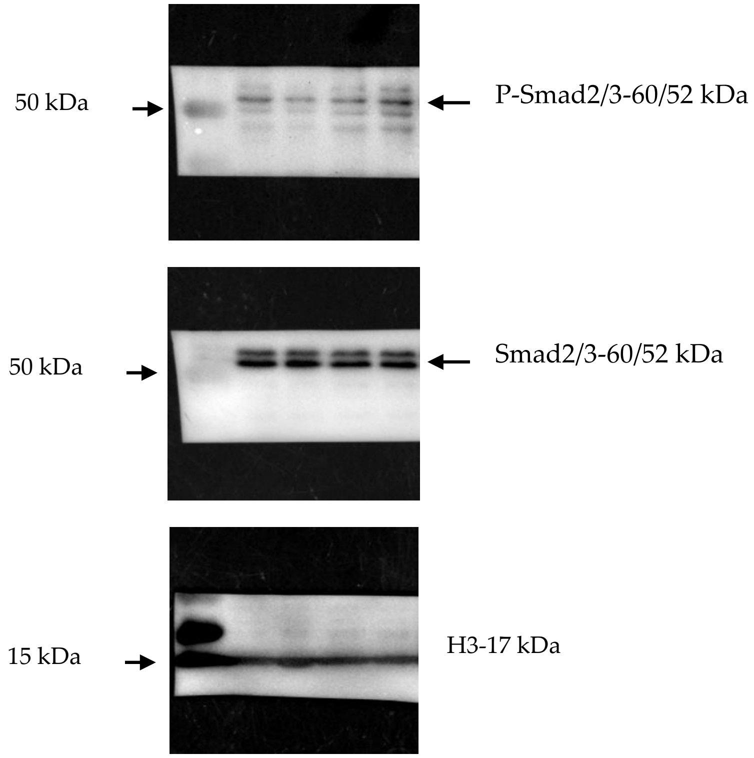

Figure 6. (**A**)

	Control	NS1643	EDTA	EDTA-NS1643
P-Smad2	0.011	0.004	0.011	0.014
P-Smad3	0.112	0.074	0.095	0.129
Smad2	0.11	0.10	0.12	0.13
Smad3	0.38	0.34	0.31	0.35

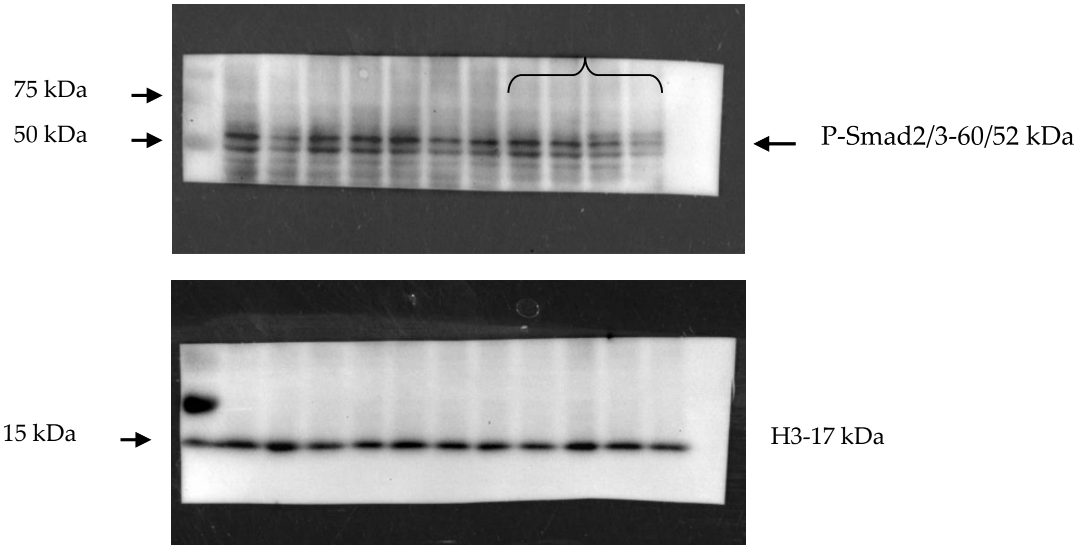

Figure 6. (B)

	Control	NS1643	Sanguinarine	Sanguinarine-NS1643
P-Smad2	0.26	0.19	0.10	0.06
P-Smad3	0.16	0.10	0.09	0.05

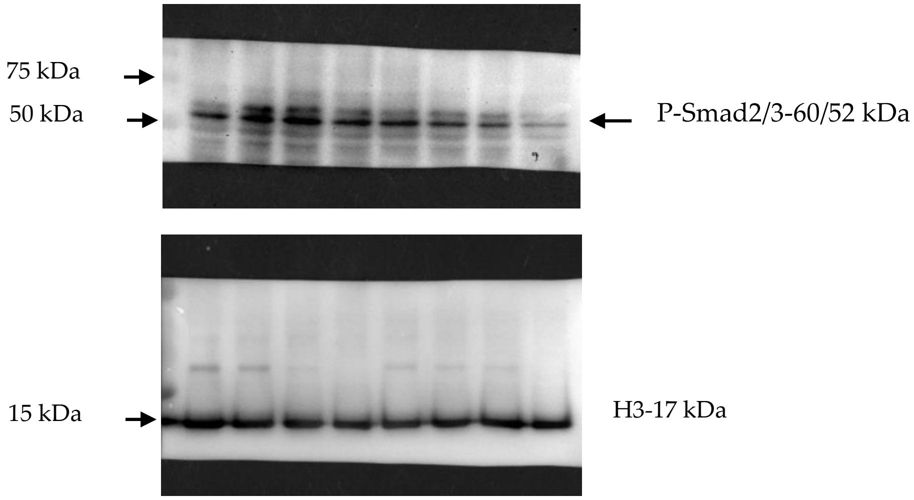

Figure 6. (C) CSA - NS1643

	Control	CSA 15 min	Control	NS1643 15 min
P-Smad2	0.015	0.015	0.015	0.006
P-Smad3	0.089	0.080	0.109	0.033

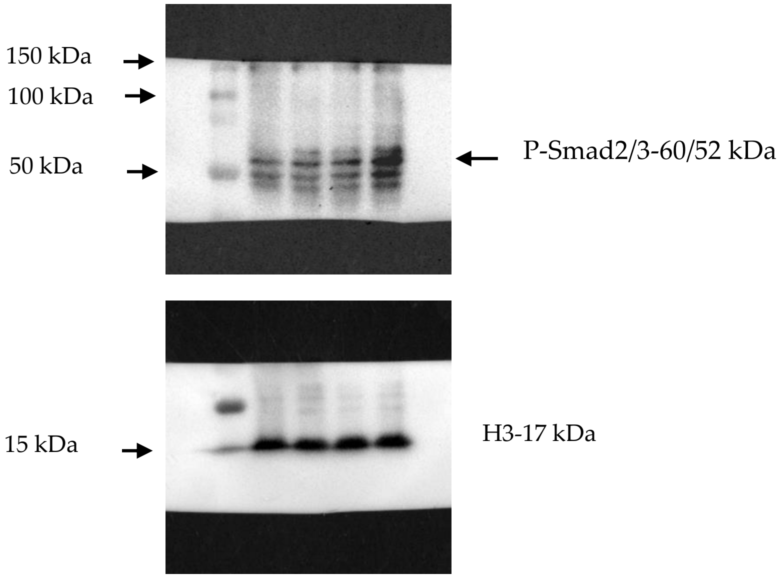

Figure 6. (**C**)NS1643+CSA

	Control	NS1643-CSA 15 min
P-Smad2	0.055	0.098
P-Smad3	0.034	0.054

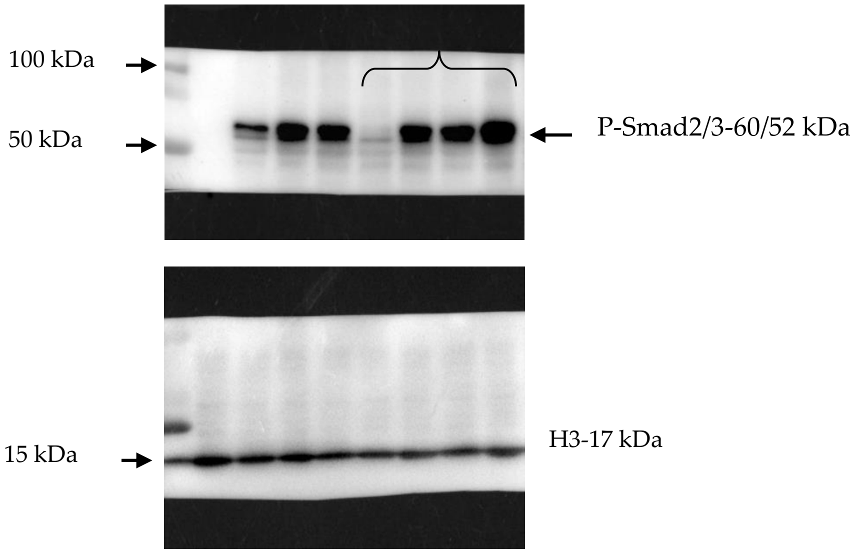

Figure 6. (**C**)TGFβ1

	Control	TGFβ 15 min
P-Smad2	0.044	0.881
P-Smad3	0.079	0.672

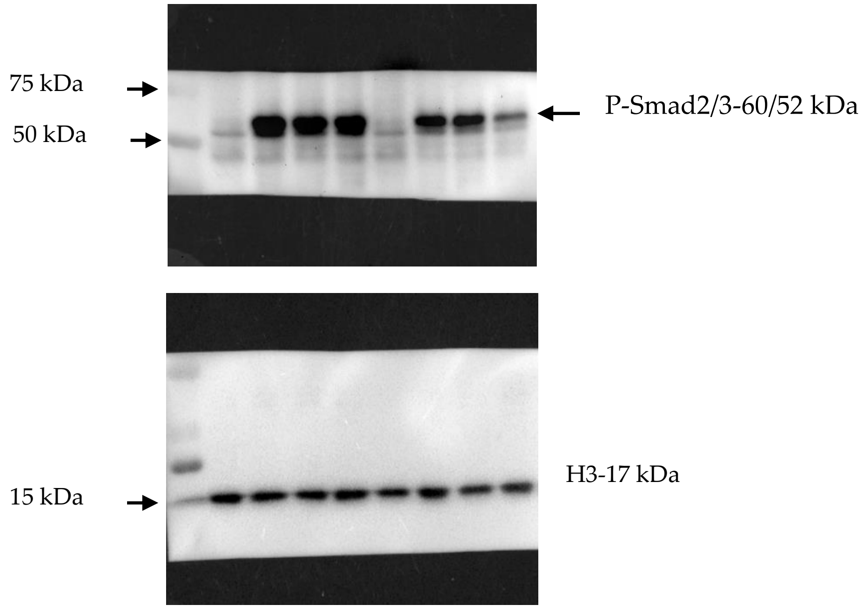

Figure 6. (**C**)NS1643+TGFβ1+CSA – NS1643+TGFβ1

	Control	NS1643-TGFβ1 -CSA 15 min	Control	NS1643-TGFβ1 15 min
P-Smad2	0.034	0.612	0.009	0.462
P-Smad3	0.123	0.524	0.070	0.124

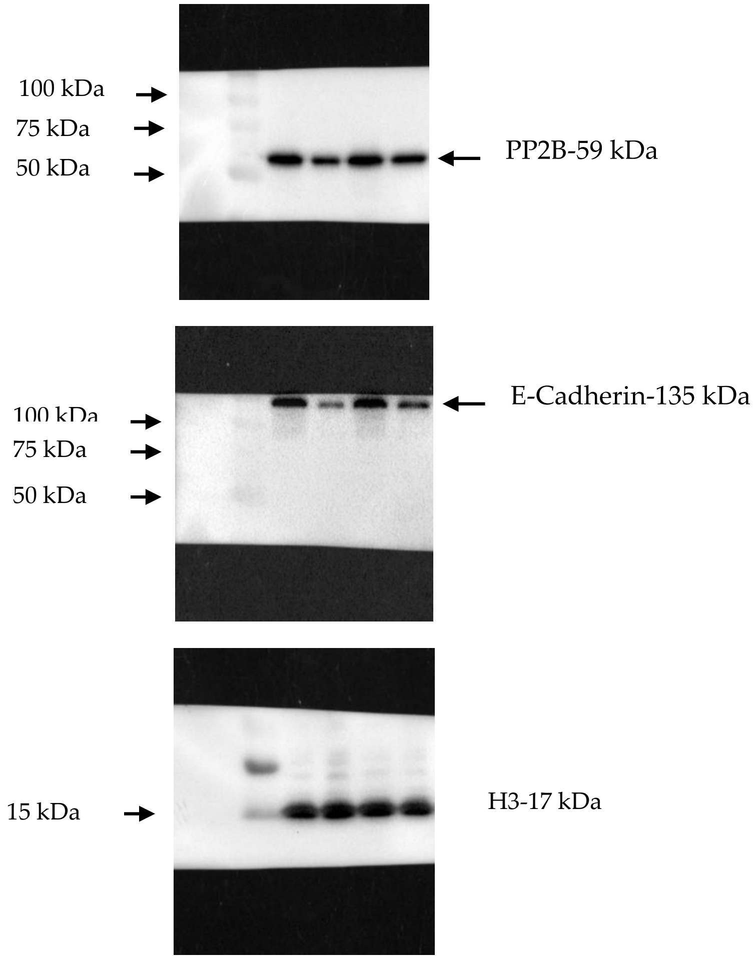

100 kDa
75 kDa
50 kDa
PP2B-59 kDa
100 kDa
75 kDa
50 kDa
E-Cadherin-135 kDa
15 kDa
H3-17 kDa

Figure 7. (E)

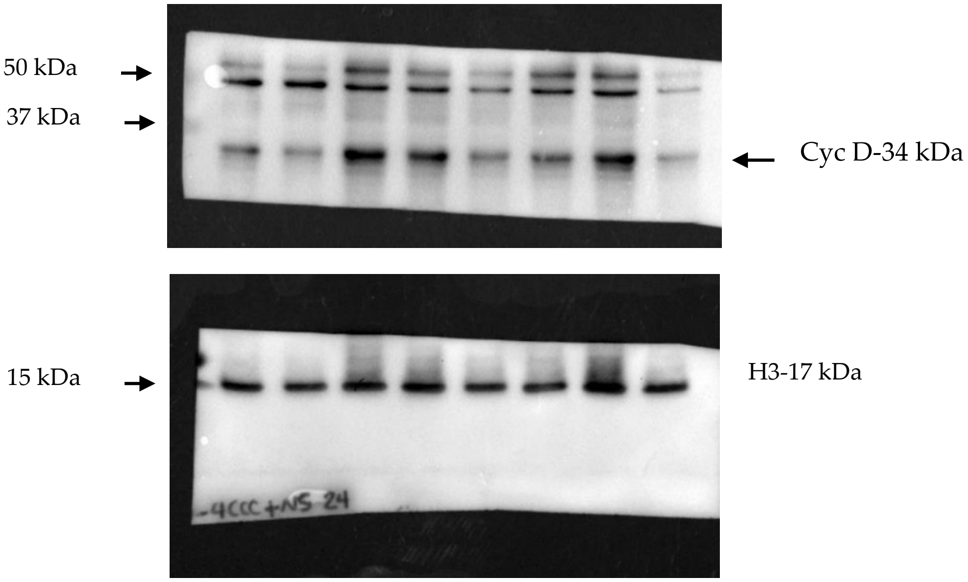

Supplementary Figure 1. (A)

	HT29-DMSO	HT29-NS1643	SW480-DMSO	SW480-NS1643	FET-DMSO	FET-NS1643
Cyc D	0.52	0.35	1.22	0.68	0.90	0.23

hERG1a – 135 kDa Alomone

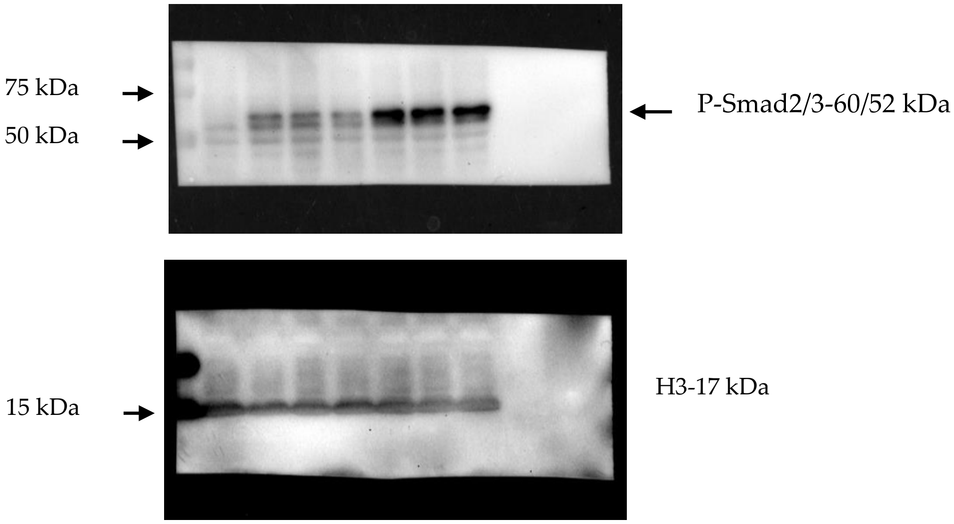

Supplementary Figure2. **(A)**

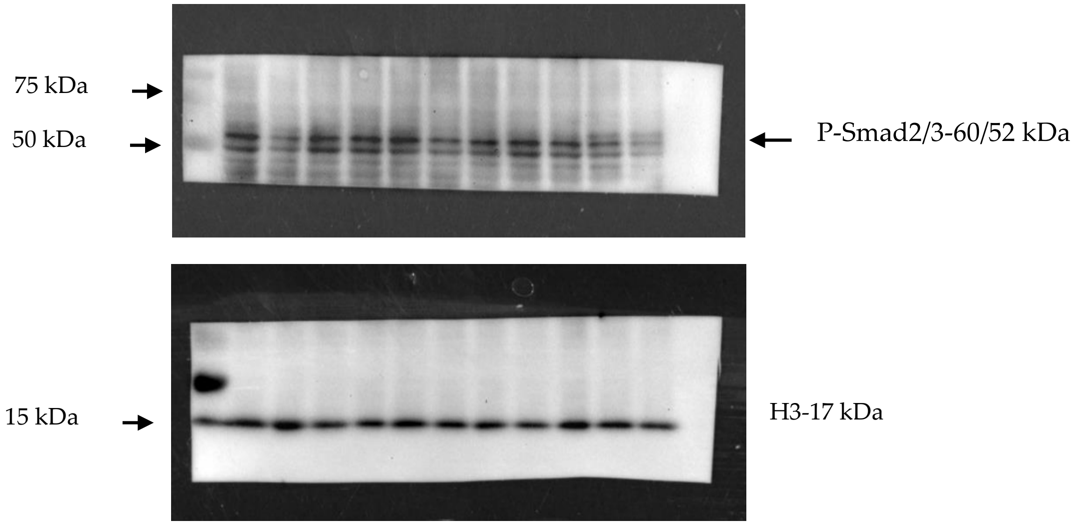

Supplementary Figure2. **(B) and (E)**

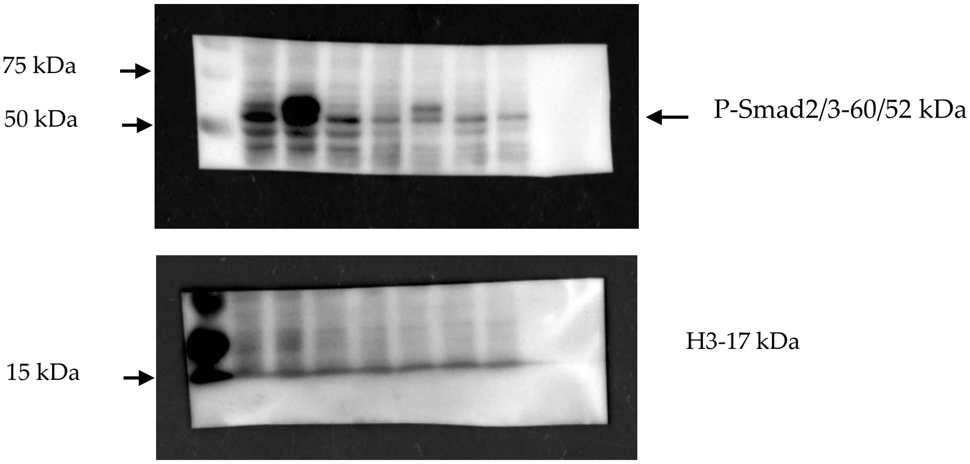

Supplementary Figure2. **(F)**
