# Supplementary material for: Molecular Activation of the Kv11.1 Channel Reprograms EMT in Colon Cancer by Inhibiting TGFβ Signaling via Activation of Calcineurin
Source: Cancers (Basel). 2021 Nov 30;13(23):6025. doi: 10.3390/cancers13236025 (PMC8656647; doi:10.3390/cancers13236025)
Supplement: Supplementary file 1 [file cancers-13-06025-s001.zip › cancers-1434748 supplementary Figure S2 and S3.pdf]

cancers

MDPI

Supplementary Materials: Molecular Activation of the Kv11.1 Channel Reprograms EMT in Colon Cancer by Inhibiting TGFβ Signaling via Activation of Calcineurin

Najmeh Eskandari, Vitalyi Senyuk, Jennifer Moore, Zane Kalik, Qiyue Luan, Ian Papautsky, Arfa Moshiri, Maurizio Bocchetta, Seyed Alireza Salami, Shahrbanoo Oryan and Saverio Gentile

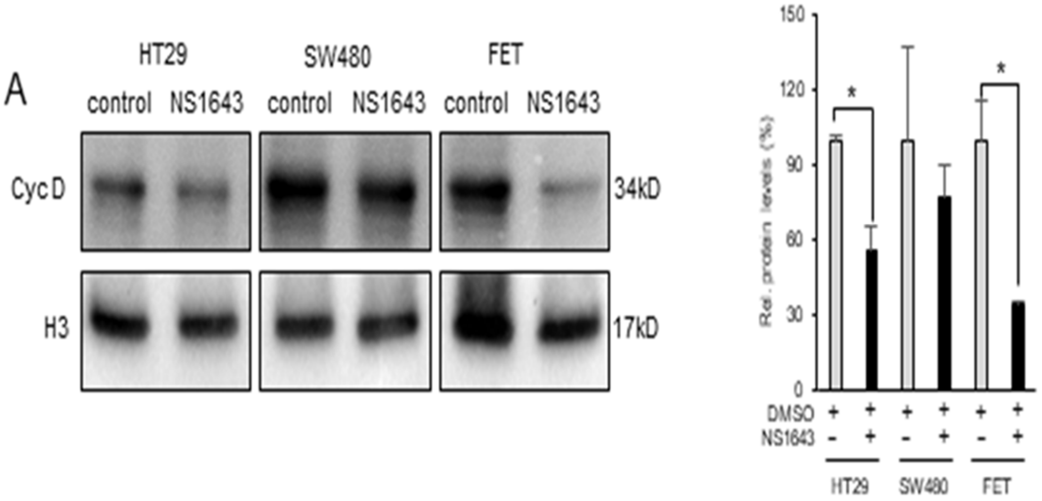

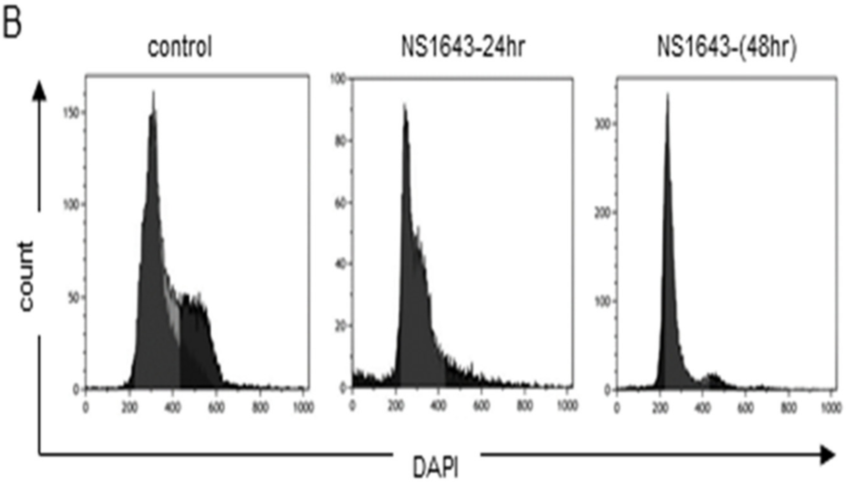

	G_1/G_0	S	G_2/M
Control	55.16%	15.36%	29.49%
24 NS1643	47.65%	39.80%	12.55%
48 NS1643	76.83%	7.41%	15.76%

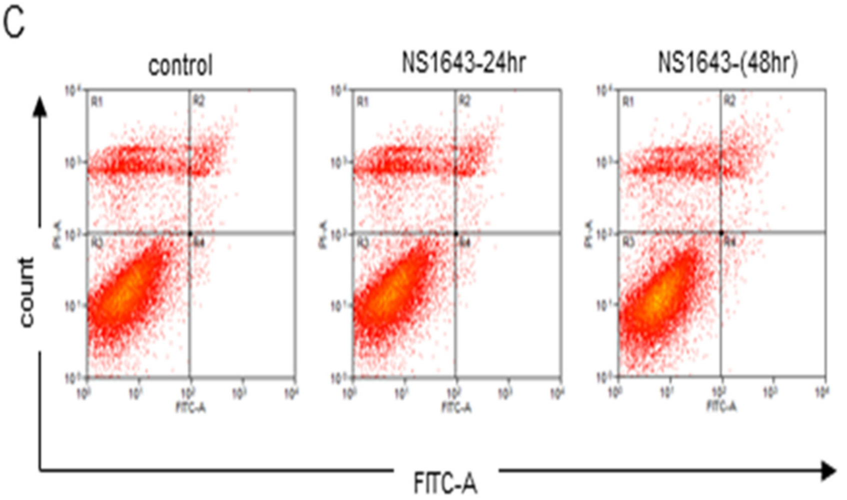

	R1	R2	R3	R4
Control	13.76%	3.10%	82.79%	0.35%
24 NS1643	12.70%	3.23%	83.46%	0.60%
48 NS1643	12.86%	2.67%	83.89%	0.58%

Supplementary Figure S2. (**A**) Western blot analysis showing expression of cyclin D in HT29, SW480 and FET cells treated with NS1643 (50μM) for 24hr. Bar graphs indicate quantification of the experiments in A. (n=3; *P<0.001). (**B**) Cell cycle analysis by Fluorescence Activated Cell Sorting (FACS) in HT29 before and after NS1643 for 24 or 48 hrs. (**C**) Cell death analysis by Annexin V detection via FACS in HT29 before and after NS1643 treatment for 24 or 48hrs.

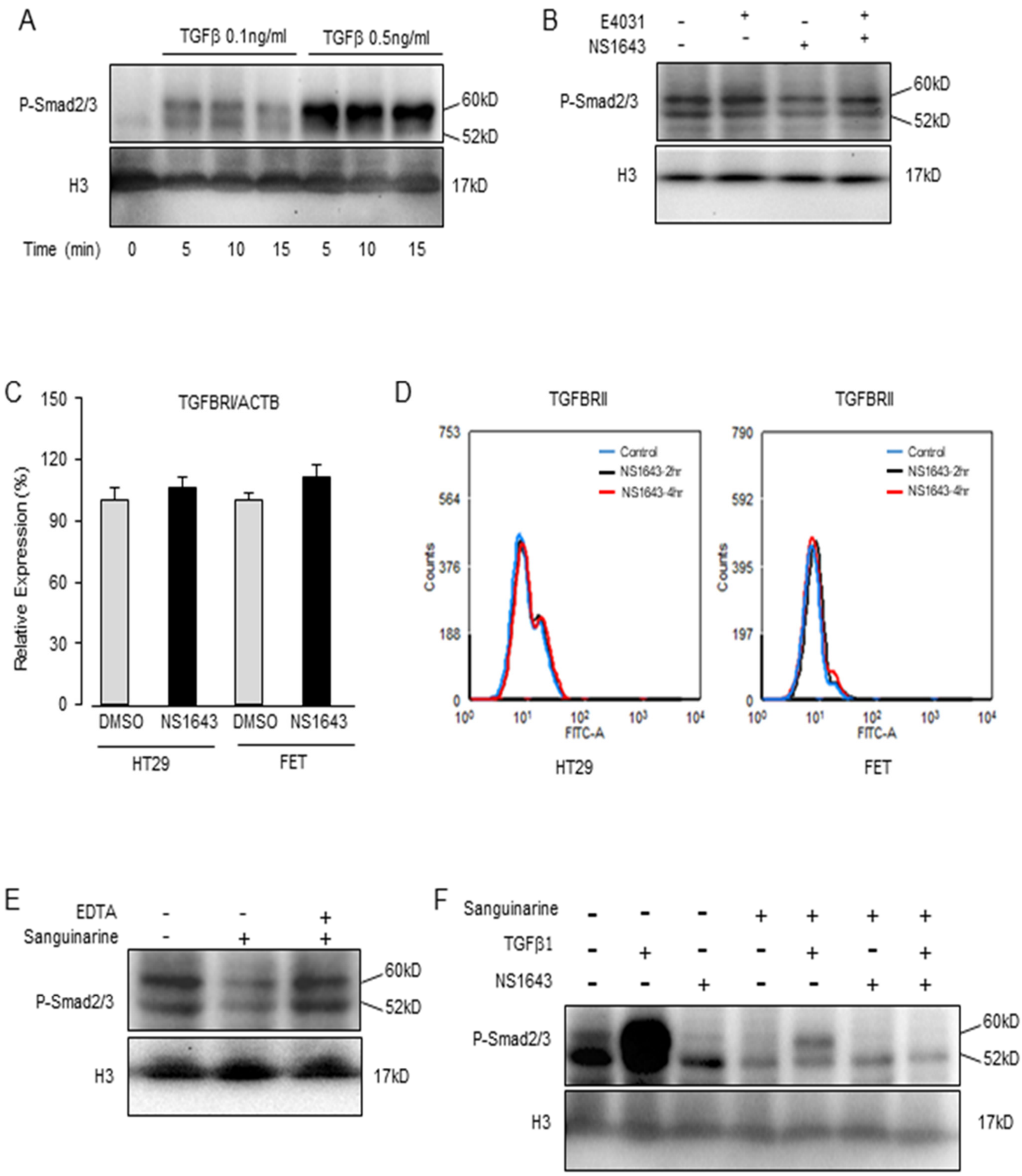

Supplementary Figure S3. (**A)** Western blot showing the effect of different concentrations of TGFβ1 at different time points as indicated on Smad2/3 phosphorylation level in HT29 cells**. (B)** Western blot showing the effect of NS1643 (50µM; 2hr) alone or with the Kv11.1 channel blocker E4031 (10µM; 16hr). (**C)** Bar graph showing the effect of NS1643 (50µM) on mRNA expression level for TGFBRI or (**D)** TGFBRII by flow cytometry in HT29 before and after NS1643 treatment (50µM) for different time points. (**E)** Western blot showing the effect of EDTA (10mM) alone, sanguinarine (1µM) alone or EDTA + sanguinarine for 2hr on R-Smads phosphorylation level in HT29. (**F)** Western blot showing the effect of sanguinarine (1µM), TGFβ1 (2ng/ml) or NS1643 (50 µM) alone in different combination as indicated on R-Smads phosphorylation level in HT29 cells.
